# Characterization and Sodium Cations Sorption Capacity of Chemically Modified Biochars Produced from Agricultural and Forestry Wastes

**DOI:** 10.3390/ma14164714

**Published:** 2021-08-20

**Authors:** Agnieszka Medyńska-Juraszek, María Luisa Álvarez, Andrzej Białowiec, Maria Jerzykiewicz

**Affiliations:** 1 Institute of Soil Sciences and Environmental Protection, Wroclaw University of Environmental and Life Sciences, 53 Grunwaldzka Str., 50-357 Wrocław, Poland; 2 Department of Geological and Mining Engineering, Universidad Politécnica de Madrid, 28003 Madrid, Spain; marialuisa.alvarez@upm.es; 3 Department of Applied Bioeconomy, Wrocław University of Environmental and Life Sciences, 37a Chełmońskiego Str., 51-630 Wrocław, Poland; andrzej.bialowiec@upwr.edu.pl; 4 Faculty of Chemistry, Wroclaw University, 14 Joliot-Curie St., 50-383 Wrocław, Poland; maria.jerzykiewicz@chem.uni.wroc.pl

**Keywords:** biochar, modification, sodium, sorbent, saline water, soil

## Abstract

Excessive amounts of sodium cations (Na^+^) in water is an important limiting factor to reuse poor quality water in agriculture or industry, and recently, much attention has been paid to developing cost-effective and easily available water desalination technology that is not limited to natural resources. Biochar seems to be a promising solution for reducing high loads of inorganic contaminant from water and soil solution, and due to the high availability of biomass in agriculture and forestry, its production for these purposes may become beneficial. In the present research, wheat straw, sunflower husk, and pine-chip biochars produced at 250, 450 and 550 °C under simple torrefaction/pyrolysis conditions were chemically modified with ethanol or HCl to determine the effect of these activations on Na sorption capacity from aqueous solution. Biochar sorption property measurements, such as specific surface area, cation exchange capacity, content of base cations in exchangeable forms, and structural changes of biochar surface, were performed by FTIR and EPR spectrometry to study the effect of material chemical activation. The sorption capacity of biochars and activated carbons was investigated by performing batch sorption experiments, and adsorption isotherms were tested with Langmuir’s and Freundlich’s models. The results showed that biochar activation had significant effects on the sorption characteristics of Na^+^, increasing its capacity (even 10-folds) and inducing the mechanism of ion exchange between biochar and saline solution, especially when ethanol activation was applied. The findings of this study show that biochar produced through torrefaction with ethanol activation requires lower energy demand and carbon footprint and, therefore, is a promising method for studying material applications for environmental and industrial purposes.

## 1. Introduction

Due to the global climate change and intensive irrigation of arable soil with saline waters, the problem of soil salinization has increased rapidly in the last decade. An extensive are of arable soils in many regions of the world has been affected by salinization, and it is estimated that approximately 33% of irrigated lands and 20% of total cultivated lands have been affected [1], thus causing a decline in soil productivity [2]. The problem is mostly observed in the arid and semi-arid climatic zones; however, due to the water shortage and human activities such as soil mineral fertilization, the use of sewage sludge, or application of salts for road de-icing during the winter season [3], the problem of soil salinization occurs locally even in more humid regions. Snow melts and rainfall in temperate climate zone are beneficial for diluting soil solution and minimizing the salt stress problem in plants. In arid climates, the excess of salts is removed from soil by irrigation; however, this technique requires ample availability of quality water resources, which is often limited in high salinity-affected areas, and it creates a practical constraint for their implementation [4]. One of the most important contaminants limiting the reuse of saline water is the excessive amount of sodium (Na^+^) and various techniques has been developed for saline water remediation [5]. The use of calcium-containing minerals, such as gypsum and calcium chloride or sulfuric acid [6,7], is very common. However, these methods are considered very expensive, environmentally unfriendly, and energy demanding; therefore, the pre-treatment of saline water before applying these methods can be deployed to reduce total desalination costs [8]. Finding an efficient and inexpensive sorbent for removing ions causing salinity from water resources is challenging. Recently, much attention has been paid to bio-waste-based materials, which is considered a green technique, offering twin benefits of waste load reduction and land and water reclamation [4]. The agricultural residues contribute as an easily available and important source of materials to be used as an environmental catalyst for the removal of contaminants [9]. Biochar obtained through the torrefaction/pyrolysis of agricultural wastes has been studied because of its high efficiency for adsorption of different inorganic pollutants [10] in soil and aqueous solutions [11,12]. Biochar seems to be a very promising material for water desalination, promoting the removal of excessive amounts of Na^+^ from solution by exchanging it with calcium, magnesium and potassium cations present on biochar surface in large amounts [4]. In general, various mechanisms of the biochar-Na^+^ sorption have been described, suggesting that physical sorption on biochar porous structure [5], preferential sorption, and ion sorption or Na^+^ exchange with biochar-borne Ca and Mg ions can all be responsible for the sorption process on biochar material [13]. In previous studies, it has been reported that the incorporation of biochar into salt-affected soil alleviated salinity stress in crop plants because of its high salt (Na^+^) potential [14]. From the practical point of view, when choosing a proper biochar based on its properties, e.g., specific surface area, cation exchange capacity, functional groups or mineral content, it is very important to match the proper selective material for cation sorption. Biochar produced from non-woody feedstock, such as manures and plant residues, is richer in nutrients, has a higher pH, and has less stable carbon than biochar produced from lignocellulose feedstock, such as wood [15,16]. Surface sorption mainly by oxygen functional groups or Na^+^ exchange with biochar-derived Ca and Mg ions are dominant mechanisms of sodium immobilization on biochar. These properties can be optimized through the appropriate designing of the torrefaction/pyrolysis process and careful selection of the biomass for thermochemical conversion. The presence of mineral (ash) components (e.g. carbonates, phosphates, or oxides) can pose both positive and negative effects on the supportive removal of contaminants where different sorption properties can be obtained during biochar deashing [17,18]. In terms of biochars derived from crop residues, e.g., straw or husk, the chemical pre-treatment might be necessary to remove excess amounts of Na^+^ [19], as part of the Na that exists in chlorides, carboxylates, or carbonates in biochar can be easily leachable, contributing to the salinization process [20]. Since water leaching is not efficient in the removal of organic compounds from biochar, acid or alcohol washing can be a more successful alternative [21]. Biochar, produced through the pyrolysis of inexpensive agricultural and forest residues, has been widely used as an alternative low-cost adsorbent to treat environmental pollution [22]. Many different human activities can increase salt pollution in surface water and drinking water resources and considering the growing problem with soil and water salinization, the prospect of biochar application for removal of sodium salts necessitates further and detailed studies.

The purpose of the study was to investigate the efficiency of Na^+^ removal from aqueous solution by three biochars derived from agricultural and forestry waste (sunflower husks, wheat straw and pinewood) with (ethanol, HCl) and without chemical modification (ethanol, HCl) and to recommend the most effective procedure of chemical modification of biochar with beneficial physiochemical characteristics to reduce Na^+^ from the solution. Recently, more attention has been paid to saline soils and different benefits of biochar in minimizing plant salt stress, explained mainly by indirect mechanisms of soil property improvements, e.g., increased content of water in soil. The knowledge about the direct mechanisms of salt sorption on biochar surfaces is less recognized. This paper describes possible mechanisms of sodium sorption on biochar, studying the efficiency of Na^+^ sorption from solution and showing possible new applications of the material. 

## 2. Materials and Methods

### 2.1. Biochar Preparation and Activation

Three types of biochar were used: wheat straw biochar (WSBC), sunflower biochar (SBC) and pinewood chips biochar (PBC), obtained as waste from agricultural and forestry activities, produced under pyrolytic conditions, respectively, at 550, 450 and 250 °C with a heating rate of (10–25 °C·min^−1^) and residence time of 1 h. All biochar samples were air-dried, ground, and sieved through a 2 mm mesh prior to all the experiments. Three chemical treatments were tested: non-modified biochar, HCl-washed biochar, and ethanol-washed biochar. To produce ethanol- and HCl-modified biochars, the procedure described by Dietrich et al. [21] was performed. Briefly, 5 g of each biochar was washed with either 0.1 M ethanol or 0.1 M HCl at a ratio of 1:9. The HCl-char and ethanol-char mixture was shaken for 24 h at 30 r.p.m. using the rotary shaker Multi RS-60 (Biosan, Saratoga Springs, NY, USA). Both types of biochar were then vacuum-extracted from solution and dried at 80 °C until constant weight. To remove residual HCl solution, the HCl-washed biochar was treated with pH-adjusted (NaHCO_3_, pH of 7.5) deionized water before drying. All determinations were performed in triplicate.

### 2.2. Biochar Analysis

The Brunauer–Emmett–Teller (BET) surface area, cation exchange capacity (CEC), pH in deionized water, CNHSO elemental composition, ash, the total contents and exchangeable cations content of Ca, Mg, K and Na were determined to describe properties of the materials. The Brunauer–Emmett–Teller (BET) surface area was determined using a specific surface area analyzer Gemini VII 2390 Series (Micrometrics, Norcross, GA, USA). Exchangeable cations (Ca^2+^, Mg^2+^, K^+^, Na^+^) were determined according to the modified method described by Munera-Echeverri et al. [23] and analyzed on a Microwave Plasma-Atomic Emission Spectrometer MP-AES 4200 (Agilent Technologies, Santa Clara, CA, USA). Cation exchange capacity was calculated as a sum of base cations. The pH values were measured at a ratio of 1:5 (*w/v*) in deionized water after the sample was shaken for 1h at 130 rpm with a calibration check pH meter (Mettler-Toledo, Graifensee, Switzerland). The ash content was determined by weight loss after combustion at 750 °C for 6 h in a muffle furnace according to ASTM D7348-13 [24]. The elemental composition was analyzed on the CHNS Vario EL Cube analyzer (Elementar, Langenselbold, Germany). To determine changes of chemical composition and biochar structure after activation with agents, Fourier Transform Infrared Spectra (FT-IR) and Electron Paramagnetic Resonance spectroscope (EPR) analyses were performed. Biochar samples were dried in an oven drier at 60 °C for 6 h to prepare pellets for FT-IR analysis. FT-IR analysis of biochar samples were recorded using a Bruker Vertex 70 FT-IR spectrometer (Bruker, Karlsruhe, Germany) using standard KBr pellets–about 1 mg sample for 400 mg of KBr. Electron Paramagnetic Resonance (EPR) spectra were obtained with Bruker Elexsys E500 spectrometer (Bruker, Billerica, MA, USA) equipped with NMR teslameter (ER036TM) (Bruker, Karlsruhe, Germany) and frequency counter (ER 41 FC) (Bruker, Karlsruhe, Germany) at room temperature. For quantitative measurements double rectangular cavity resonator (ER 4105DR) (Bruker, Karlsruhe, Germany) operating at the mode TD104 was applied. In one cavity standards of radical concentration were placed while the analysed sample in the second cavity. After tuning, the spectra were recorded separately for both cavities without changing any of the measurement parameters. X-band spectra were measured at microwave power of 20 mW, modulation amplitude of 1 G. For measurements the solid samples (about 20.0 mg) were placed in quartz tubes of 5 mm outer diameter. The Li/ LiF standard was used for g-parameter calibration (g = 2.00223). As standards of spin concentration Pahokee peat standard humic acid (1S103H) and Leonardite standard humic acid (1S104H) extracted and distributed by International Humic Substances Society (IHSS) [25], and additionally, Bruker alanine pills were used as standards of spin concentration analysis. For quantitative measurements, a double rectangular cavity resonator–(ER 4105DR) (Bruker, Karlsruhe, Germany) operating at the mode TD104 was used. The standards of the radical species concentration were placed in the first cavity and the analyzed sample in the second cavity. After tuning, the spectra were recorded for both cavities separately without changing any of the measurement parameters.

### 2.3. Sodium Adsorption on Biochar Experiment 

For the adsorption experiment, sodium chloride (NaCl), ACS reagent, ≥99.0% (Sigma Aldrich, Saint Louis, MO, USA) was diluted in Milli-Q^®^ ultrapure water (Merckmilipore, Burlington, MA, USA) to obtain 1%; 0.2%; 0.5% and 1.0% NaCl solutions. For that, 1 g of each biochar (WSBC, SBC and PBC) was weighed in a falcon tube for every 10 mL of NaCl solution. The scheme of the experiment and tested variants of prepared samples were listed in Appendix A, Table A1. The concentrations of NaCl in the solution imitated the amounts of sodium delivered to soil during different activities, mainly soil fertilization, field irrigations with low-quality waters, road run offs and saline waters deposited in the environment from mining activities, where concentrations of sodium can reach up to 5%. The content of potential exchangeable Na^+^, Ca^2+^, Mg^2+^ and K^+^ in NaCl solutions used in the sorption experiment is presented in Appendix A Table A2. Biochar samples were shaken for 24 h, and approx. 1 mL of solution was collected after each treatment. Sodium concentrations were analyzed on Microwave Plasma-Atomic Emission Spectrometer MP-AES 4200 (Agilent Technologies, Santa Clara, CA, USA), after sample filtration on Munktell No. 2 filters (Ahlstrom Munksjö, Helsinki, Finland) in dilutions of 1:10 or 1:100 for torch protection. 

### 2.4. Calculations and Statistical Analysis

Sodium sorption batch experiments were performed in triplicate. The data are presented as the mean values with the relative standard deviation (RSD). Student’s *t*-tests were used to test for significant differences in element content between non-modified biochars and biochars modified with EtOH and HCl (*p* < 0.05). The obtained data were compiled using Microsoft Excel 2016 and Statistica Statsoft 13.3. FT-IR spectra were performed for absorbance to simplify the interpretation of intensity ratios. Characteristic areas of the spectra were integrated using data analysis and graphing software OriginPro2019 (OriginLab Corporation, Northampton, MA, USA.) The percentages were calculated according to the whole spectrum area. The isotherms of Na adsorption on each type of biochar were determined. Langmuir’s (Equation (1)) and Freundlich’s (Equation (2)) models were tested.

(1) $$qe=\left(\frac{\left(qm\cdot b\cdot Ce\right)}{\left(1+b\cdot Ce\right)}\right)\;$$

where:

*qe*—the equilibrium (instantaneous) amount of adsorbed Na ions on units of biochar mass, mg/g,
*qm*—the maximum amount of adsorbed Na ions on units of biochar mass, mg/g, to form a complete monolayer on the surface,
*Ce*—equilibrium concentration of Na ions, mg/L,
*B*—Langmuir adsorption constant related to the energy of adsorption, L/mg.

(2) $$qe=Kf\cdot {Ce}^{\left(\frac{1}{n}\right)}\;$$

where:

*qe*—the equilibrium (instantaneous) amount of adsorbed Na ions on units of biochar mass, mg/g,
*Kf*—Freundlich adsorption constant, mg∙g^–1^∙(L∙mg^–1^)^(1/*n*),
1/*n*—empirical constant: heterogenicity coefficient.

For the determination of isotherms parameters nonlinear regression analysis was done. The regression analysis was done using the Statistica 13 software (StatSoft, Inc., TIBCO Software Inc., Palo Alto, CA, USA). For the validation of model parameters, the determination coefficient (R^2^) was calculated at the statistical significance (*p* < 0.05). After that both models were compared with application of the Akaike Information Criterion (AIC) to indicate the simplest model matching to raw data similarly. AIC was evaluated according to the following Equation (3):

(3) $$AIC=n\cdot ln\left(\sum_{i=1}^{n}e_{i}^{2}\right)+2\cdot K$$

where:

*AIC*—value of Akaike analysis,
*n*—the number of measurements,
*e*—the value of the rest of the model for a particular measurement point,
*K*—the number of regression coefficients, including an intercept in the model.

Generally, models with a larger number of predictors are more accurate but tend to over-fitting. The AIC approach can be used to preserve good accuracy and a low number of predictors in the compared models. When models for a particular variable are compared, a model with a lower AIC is better.

## 3. Results and Discussion

### 3.1. Biochar Sorption Properties

Previous studies have shown that the adsorption of Na^+^ is related to cation exchange capacity (CEC) of biochar as the CEC value is closely related to the concentration of oxygenated functional groups contributing to the sorption process of different cations [5]. In terms of studied biochars, CEC was strongly related to feedstock type used during the pyrolysis process, and wheat straw biochar presented the best potential for cation sorption, having the highest specific surface area, cation exchange capacity and pH (Table 1 and Table 2). However, very high content of exchangeable Na^+^–27.01 cmol(+)/kg in WSBC, compared with 1.13 cmol(+)/kg in SBC and 1.99 cmol(+)/kg PBC–suggested that WSBC can become an Na^+^ donor to the solution. The contribution of biochar as a donor of Na^+^ was investigated by Sarpong et al. [19], studying the effect of halophyte *Atriplex*-derived biochar application to saline soil and the process of Na^+^ leaching from biochar. The results of the study showed that biochar derived from biomass grown on saline soils contained higher amounts of Na^+^ readily exchangeable with cations in soil solution, contributing to the salinization process. The highest content of potentially exchangeable Na^+^ cations (K^+^, Ca^2+^ and Mg^2+^) was determined in PBC (Table 1), suggesting that hard-wood derived biochar will have the best abilities for salt removal via an ion exchange mechanism. 

Sample treatment with ethanol caused an almost twofold decrease in ash content and CEC in WSBC, increasing the biochar acidity and removing almost half of the exchangeable Na^+^ during this modification (Table 1). In SBC and PBC, cation exchange capacity was improved as the content of base cations (Ca^2+^, Mg^2+^ and K^+^) in exchangeable forms increased, probably due to the partial dissolution of low molecular weight organic compounds and rinsing of the excess of NH_4_^3+^ cations and surface changes [17,23]. HCl pre-treatment had very adverse effects on the tested biochars. In terms of WSBC, it did not cause significant sample de-ashing, while in PBC, almost 6% of ash was removed by acid. In SBC and PBC, the sample treatment with HCl caused an increase in CEC and acidity; however, this increase was balanced by the release of Ca^2+^ in exchangeable forms and probable decomposition of carbonates present on the biochar surface after treatment with HCl. pH is a very important property regarding the surface charges on biochar. When biochar is applied to aqueous solution for metal removal, the solution pH strongly influences its surface charge. At a solution pH of 3–7, biochars become negatively charged, which favors positively charged ion sorption [21]. At higher temperatures, the number of negatively charged groups on biochars is reduced, and those acidic modifications may have a positive effect on surface charges and occurrence of negatively charged functional groups attracting Na^+^. The changes of the surface charges were not investigated in this study; however, the results from the FTIR analysis and SSA determination showed that chemical modification caused changes on biochar surface, and depending on a feedstock type and initial pH, the material improved or had no significant effect on Na^+^ sorption on tested biochars. An increase in SSA after tested chemical-pretreatments was observed in all three biochars; however, significant differences were only determined for ethanol-treated samples. The best effect of the SSA increase was observed after ethanol treatment of WSBC, and the SSA increased from 265 to 387 g/m^2^. The increase was also significant for EtOH-SBC (from 80.5 to 93.2 g/m^2^), while no significant changes were observed for EtOH-PBC (Table 2). Comparing both methods of biochar modifications, ethanol pre-treatment was more efficient for SSA improvement compared with HCl.

### 3.2. Surface and Structural Changes of Biochar after Modifications

Generally, the content of carbon was strongly related to the temperature of the pyrolysis, and the lowest values were found for WSBC produced at 550 °C, while in the case of PBC, the temperature characteristic for torrefaction at 250 °C preserved the C content. All biochars had low nitrogen content (<0.9 %), and as expected, the lowest was in PBC (0.42%), and the highest was in SBC (0.95%). Hydrogen content was similar for all non-modified biochars, from 2.2% in non-WSBC to 2.57% in non-SBC. Surprisingly the content of sulfur in all non-modified biochars was very low (0.001–0.003%) and increased significantly (even up to 0.0425%) after biochar treatment with agents (Table 3). Biochar modifications changed the elemental composition of biochars, especially when EtOH was used as an agent. The effect of chemical activation depended also on the biochar type. EtOH treatment significantly increased content of C, N, H and S in WSBC and SBC, decreasing O content in both materials. In contrast, in PBC, EtOH treatment caused material oxidation, increasing O content from 7.58% to 23.69% and decreasing C from 89.55% to 72.36%, which could be related to the elution of low molecular weight products of torrefaction. HCl treatment was less effective, and even in the case of H where protonation was expected after HCl treatment, the elemental composition did not change significantly. The obtained BC molar ratios emphasize the presence of aromatic structural features and reduced content of O-containing polar functional groups on the biochar surface (low molar O/C ratio and polarity index) after WSBC and SBC treatment with EtOH and HCl, and opposite results and increase in polar oxygen-containing surface functional groups on PBC. 

**Figure 1:**
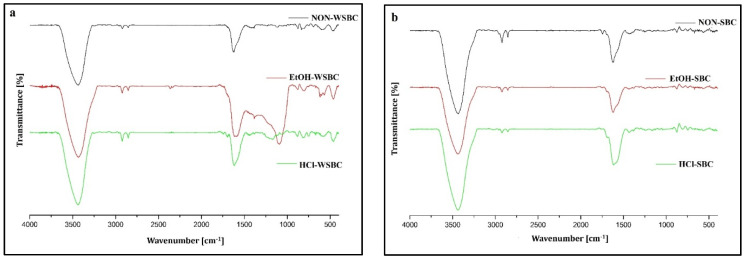

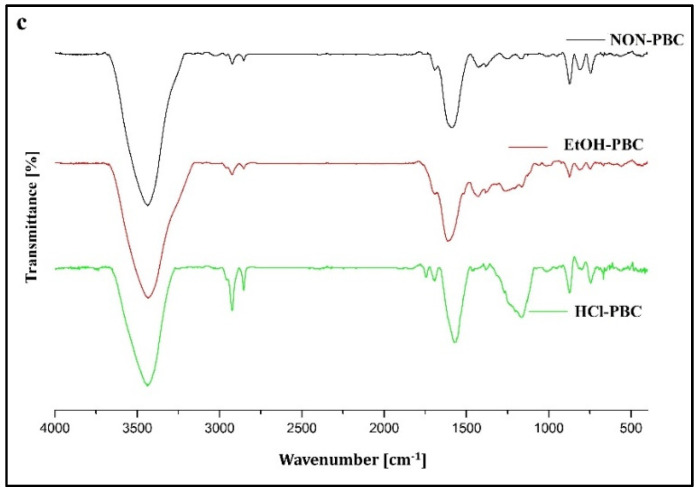
FT-IR spectra of investigated biochars: (**a**) pure (non-WSBC) and modified (EtOH-WSBC and HCl-WSBC) wheat straw biochar; (**b**) pure (non-SBC) and modified (EtOH-SBC and HCl-SBC) sunflower husk biochar; (**c**) pure (non-PBC) and modified (EtOH-PBC and HCl-PBC) pinewood biochar.

Obtained FT-IR spectra were typical for biochar material, showing bonds related to –OH (hydrogen groups) at 3400 cm^−1^, C–H (aliphatic groups) at 2950–2850 cm^−1^ and C–C (aromatic groups) at 1630 cm^−1^ and C=O (carboxylic groups) at 1624 cm^−1^ (Figure 1). The strongest peaks were observed for all three biochars at 3400 cm^−1^, showing that hydrogen groups were dominant on their surface, followed by the presence of carboxylic groups bands and weak peak of aliphatic groups (the highest in SBC). Similar FTIR spectra were obtained in a study by Rostamian et al. [8], describing rice husk biochar properties and Na sorption capacity. WSBC was also more visible compared with other biochar peaks located at 469, 803 and 1098 cm^−1^ ascribed to bending vibration, symmetric stretching, and asymmetric stretching of Si–O bonds, typically found in biochars derived from monocot plants such as grasses and cereals due to the presence of silica. In WSBC and PBC, an increase in the aliphatic C–H and aromatic C–C vibration bands and decrease in –OH vibration bands were observed after a sample treatment with EtOH and HCl; however, the effect depended on the agent type. For WSBC, treatment with EtOH significantly increased the number of carboxyl and =C=O of amides, ketones and quinones compared to non-WSBC and HCl-WSBC. Sharp peak located at 1098 cm^−^^1^ was increased after WSBC treatment with ethanol, suggesting that an alcohol agent could increase silica content of WSBC. HCl sample treatment had no significant effect on tested biochar surfaces, with the exception of PBC, where a strong carboxyl peak was indicated after acid activation. The findings of our study are in contradiction to the results of Li et al. 2014 [17], who presented that wheat straw-derived BC modified with different concentrations of HCl (1.0 mol/L and 6.0 mol/L) developed a more heterogeneous porous structure compared with untreated samples. Surprisingly, from three tested biochars, sunflower husk biochar was the most “resistant” material to chemical activation, and very similar spectra were obtained for raw and activated SBC. Functional groups present at the biochar surface are one of the crucial factors determining the physical adsorption process of Na^+^ [26,27].

Calculations of FT-IR integrated areas confirmed the variability of the observation obtained during spectra analysis. For wheat straw biochar, EtOH treatment increased the aromaticity, reducing the number of –C–H and –OH groups on its surface. For SBC, aromaticity increased after HCl treatment, while EtOH increased the number of –OH groups. In PBC, EtOH increased aromaticity, while HCl treatment decreased the number of –OH groups, increasing –C–H (Table 4). Biochars can donate, accept, or transfer electrons in their surrounding environments, which is probably the most important property of the material when concerning environmental applications, such as cation removal [28]. According to the study by Klüpfel et al. [29], different mechanisms can be distinguished between the biochars produced at different temperatures, and spectroscopic analysis provides evidence that the pool of redox-active moieties is dominated by electron-donating, phenolic moieties in the low-temperature biochars, by newly formed electron-accepting quinone moieties in intermediate-temperature biochars, and by electron-accepting quinones and possibly condensed aromatics in the high-temperature pyrolysis. Awan et al. [13] described that biochars containing highly structured, aromatic compounds that induce electronegativity in the form of delocalized *π* electrons become more electrochemically active than original feedstock. This electronegativity may effectively sorb soft base cations (e.g., Na^+^) to a greater extent than hard base cations (e.g., Ca^2+^ and Mg^2+^) due to the weak hydration and relatively large radii of soft base cations. 

EPR analysis of radicals built in the biochar matrices show that there were no structural changes to the investigated biochar during EtOH and HCl treatment (g-parameter did not change). However, a difference was observed in radical concentrations. The quenching of the radicals was especially observed for biochar originated from pinewood after HCl treatment. Non-WSBC was characterized by the lowest radical concentration, but a decrease in radical concentration was also observed in HCl-WSBC (Table 5).

EPR analysis of biochar suggests that certain chars contain radicals of semiquinone-type character and the highest concentration quinone concentrations can be obtained during intermediate to high temperature pyrolysis [28]. However, this phenomenon was not confirmed in our study as the lowest radical concentrations were determined in high-temperature WSBC.

### 3.3. Sodium Sorption Experiment

#### 3.3.1. The Mechanism of the Adsorption

**Figure A1:**
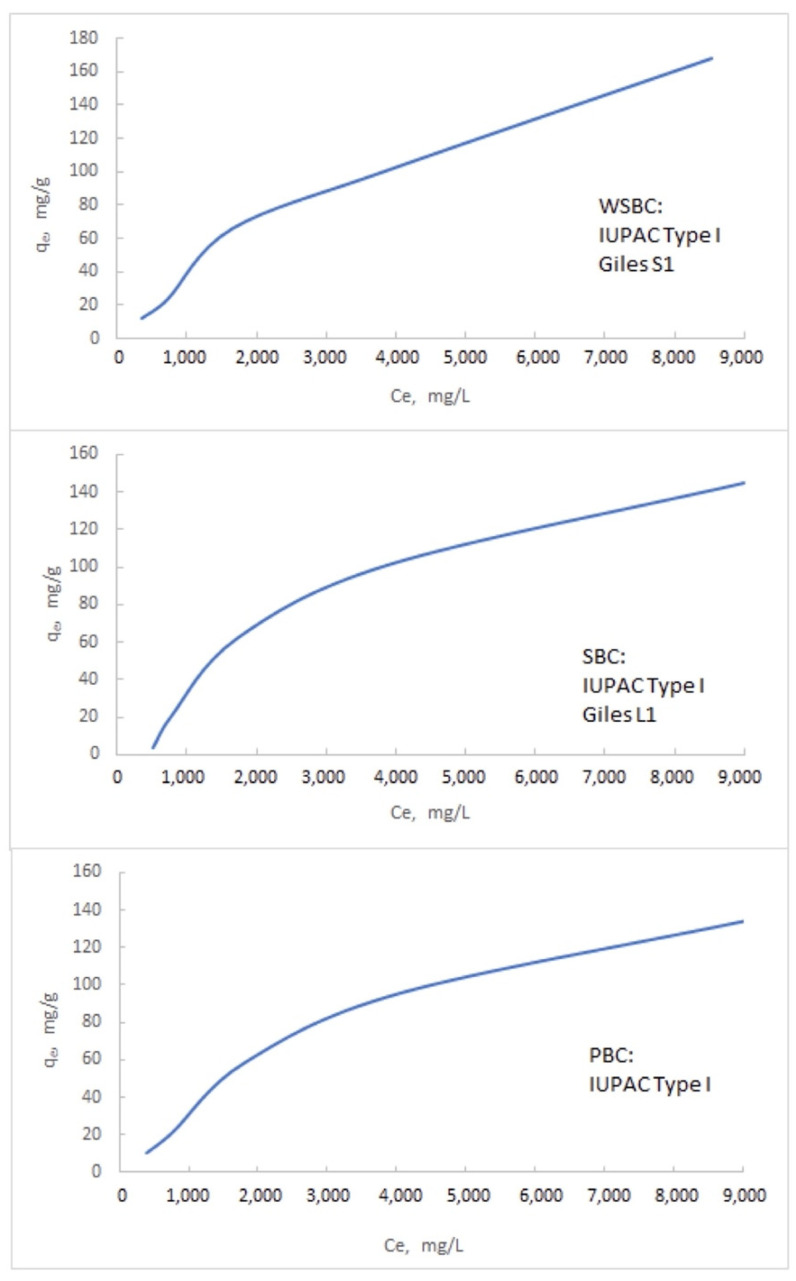
The changes of maximum amount of adsorbed Na ions on units of non-activated biochar mass, *q_m_*, mg/g depending on *C_e_*—equilibrium concentration of Na ions, mg/L. The IUPAC and Giles adsorption types of classification have been given.

**Figure A2:**
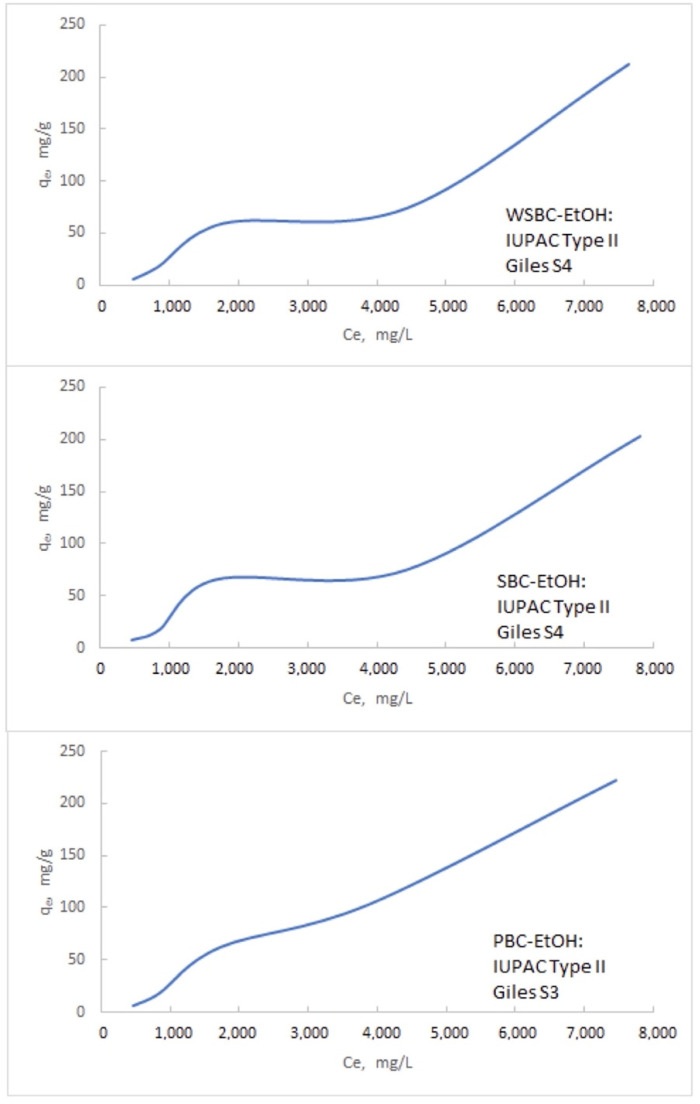
The changes of maximum amount of adsorbed Na ions on units of EtOH-activated biochar mass, *q_m_*, mg/g depending on *C_e_*—equilibrium concentration of Na ions, mg/L. The IUPAC and Giles adsorption types of classification have been given.

**Figure A3:**
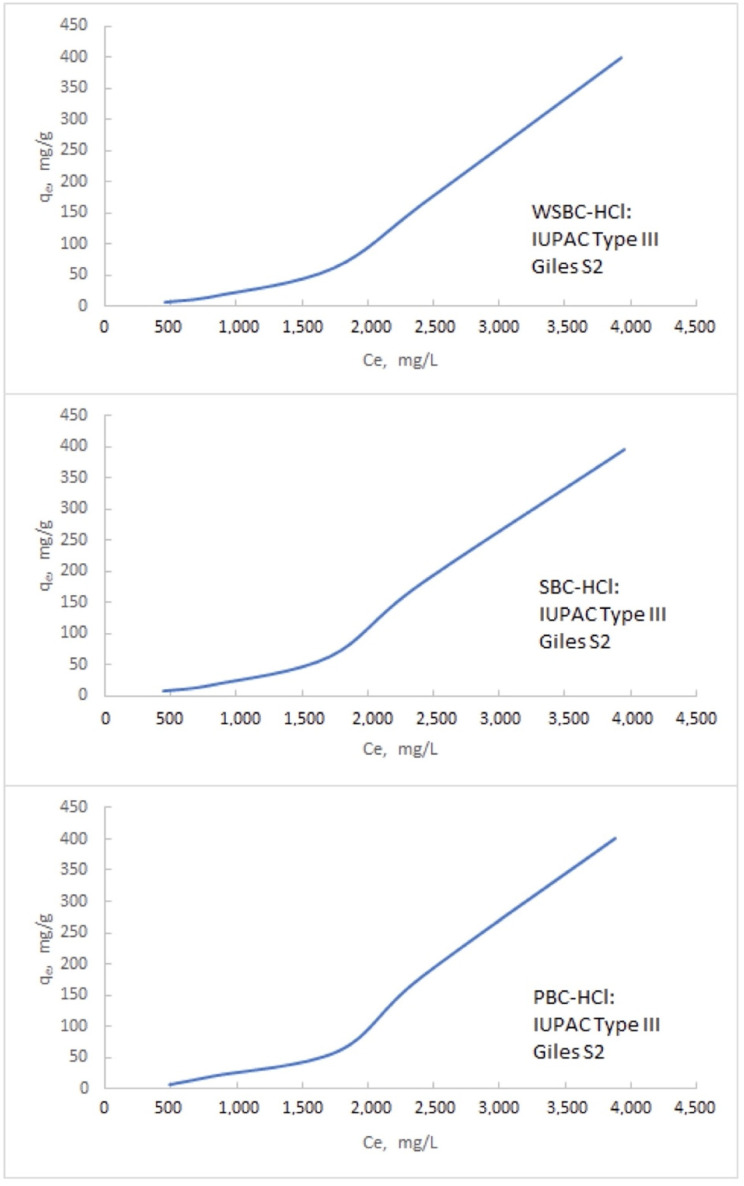
The changes of maximum amount of adsorbed Na ions on units of HCl-activated biochar mass, *q_m_*, mg/g depending on *C_e_*—equilibrium concentration of Na ions, mg/L. The IUPAC and Giles adsorption types of classification have been given.

The adsorption of Na^+^ in the analyzed types of activated and non-activated biochars had different patterns, which have been shown in the appendix (Figure A1, Figure A2 and Figure A3). The Na^+^ adsorption on non-activated PBC and SBC surfaces had the L type character according to Giles classification (Figure A1). In the case of WSBC, S-type is visible. In all the cases, the single layer of the BC surface is not fully covered by Na ions, which means that the adsorption type belongs to the first sub-class [30]. According to IUPAC classification, the adsorption curves of all biochars are characteristic of type I, which is characteristic of microporous adsorbents, for which there is a strong affinity adsorbent–adsorbent [31]. The Na^+^ adsorption on EtOH-WSBC and EtOH-SBC surfaces had clear S-type character according to the Giles classification (Figure A2). In the case of PBC, S-type is also visible. The S class includes isotherms for which, in the low equilibrium concentration range, the isotherm contains an inflection point behind which the curve increase is sharper. Adsorption, in this case, is preferred at higher concentrations. For this situation to occur, three conditions must be met: (i) the adsorptive molecule must interact with the surface of the condensed phase through interactions of only one type, (ii) have a moderate affinity for the adsorbent surface, and (iii) encounter strong competition from solvent molecules. In the case of EtOH-WSBC and EtOH-SBC, it belongs to the fourth sub-class. The third subgroup, identified for EtOH-PBC, contains isotherms for which the formation of the next layer of adsorption is observed, which is possible only in cases of physisorption [30]. According to IUPAC classification, the adsorption curves are characteristic of type II. Reversible Type II isotherms are given by the physisorption on nonporous or macroporous adsorbents. The shape is the result of unrestricted monolayer-multilayer adsorption. If the knee is sharp, Point B—the beginning of the middle, almost linear, section—usually corresponds to the completion of monolayer coverage of EtOH-WSBC and EtOH-SBC. A more gradual curvature (i.e., a less distinctive Point B) is an indication of a significant amount of overlap of monolayer coverage and the onset of multilayer adsorption, such as in the case of EtOH-PBC (Figure A2).

The Na^+^ adsorption on HCl-activated PBC, WSBC and SBC surface had clear S-type character according to the Giles classification (Figure A3). In the case of all biochars modified by HCl, the isotherms belong to the second sub-class. The second subgroup includes isotherms for which the surface saturation with adsorbate monolayer has been achieved [30]. According to IUPAC classification, the adsorption curves of all types of HCl-biochars are characteristic of type III. Type III is also characteristic of macroporous adsorbents, but in this case, the adsorbent–adsorbent interactions are weak. In the case of a Type III isotherm, there is no Point B and therefore no identifiable monolayer formation; the adsorbent–adsorbate interactions are now relatively weak, and the adsorbed molecules on Na^+^ are clustered around the most favorable sites on the surface of a nonporous or macroporous solid. In contrast to a Type II isotherm, the amount of Na^+^ adsorbed remains finite at the given concentration [31].

#### 3.3.2. The Isotherms of the Na Adsorption

**Figure A4:**
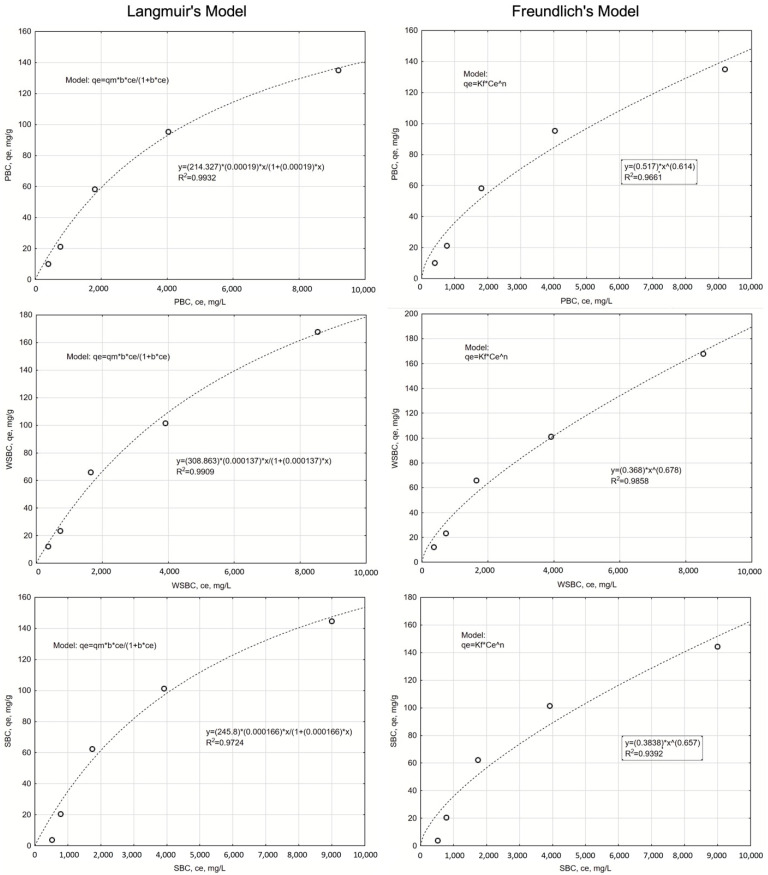
The modeling of Na adsorption of Langmuir’s and Freundlich’s isotherms on non-activated biochar, *q_e_*—the equilibrium (instantaneous) amount of adsorbed Na ions on units of biochar mass, mg/g, *C_e_*—equilibrium concentration of Na ions, mg/L. The model parameters and R^2^ values have been given.

**Figure A5:**
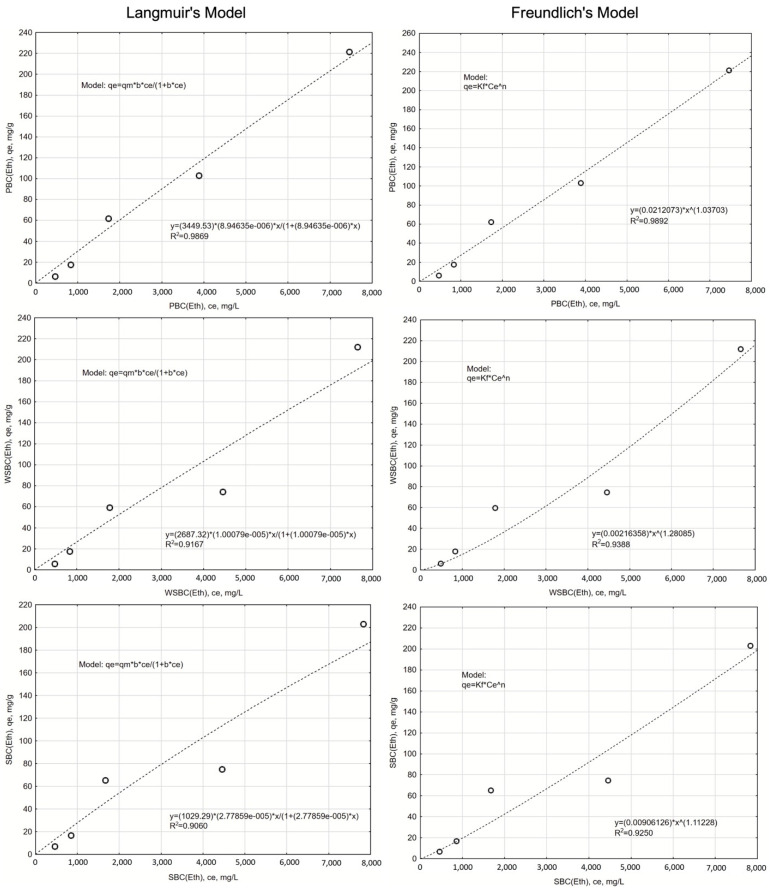
The modeling of Na adsorption Langmuir’s and Freundlich’s isotherms on ethanol-activated biochar, *q_e_*—the equilibrium (instantaneous) amount of adsorbed Na ions on units of biochar mass, mg/g, *C_e_*—equilibrium concentration of Na ions, mg/L. The model parameters and R^2^ values have been given.

**Figure A6:**
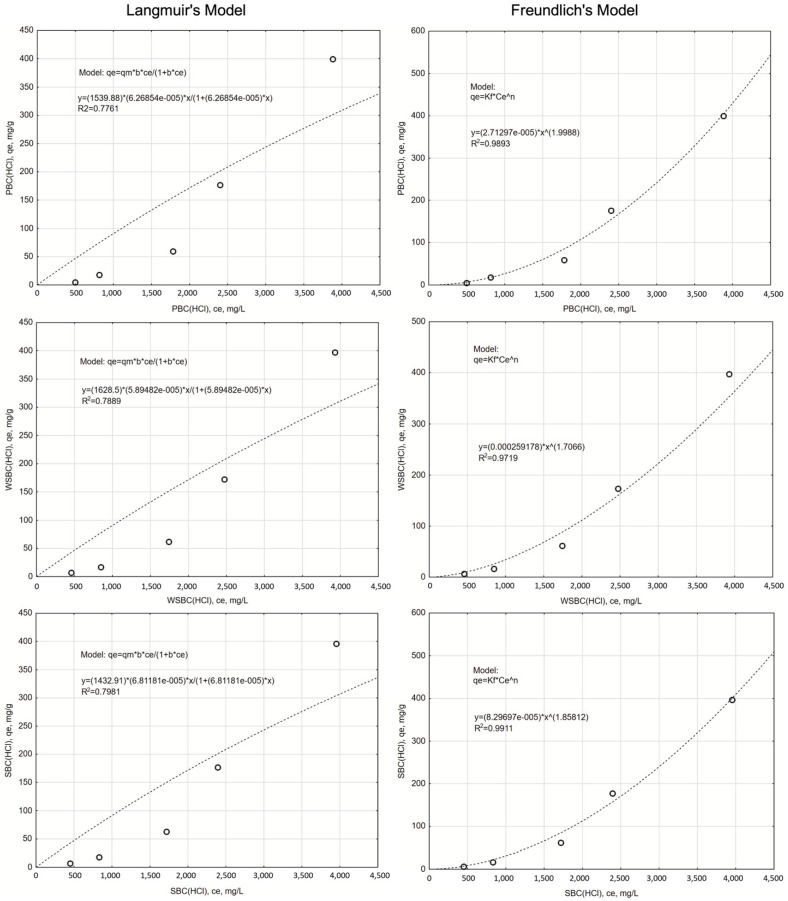
The modeling of Na adsorption Langmuir’s and Freundlich’s isotherms on HCl-activated biochar, *q_e_*—the equilibrium (instantaneous) amount of adsorbed Na ions on unit of biochar mass, mg/g, *C_e_*—equilibrium concentration of Na ions, mg/L. The model parameters and R^2^ values have been given.

For the investigation of adsorption isotherms, two models were tested: Langmuir’s and Freundlich’s. The patterns of isotherms in non-linear regression modeling are presented in Appendix B (Figure A4, Figure A5 and Figure A6). The performed non-linear regression analyses indicated very high fitting of both models to the experimental data, confirmed by high values of determination coefficients (Table 6). In the case of non-activated biochars, R^2^ is higher in Langmuir’s model. The additional parameter AIC indicates lower values than in the case of the Freundlich model, which confirms that Langmuir’s model should be preferred in the case of non-activated biochars. It has been shown that the maximum Na^+^ adsorption capacity was found in the case of WSBC, which is associated with the highest values of cation exchange capacity and specific surface area. Results showed that the biochar from high-temperature pyrolysis should be preferred. These reactions were previously explained by Klüpfel et al. [29], showing that chars produced at intermediate to high heat treatment temperatures (400–700 °C) have higher capacities to accept and donate electrons compared to low-temperature biochars, such as the tested PBC. Tan et al. [5] described mitigation of soil salinity and showed that biochar derived from lignocellulosic biomass produced at high pyrolysis temperatures developed bigger pore volumes and specific surface area, providing higher binding sites for Na^+^ ions.

Executed modeling of Na^+^ adsorption isotherms of ethanol-treated biochars indicated a significant (10-fold) increase in the maximum Na^+^ adsorption capacity (Table 7), which could be related to the elution of organic compounds with high oxygen content. The EtOH-PBC had the highest adsorption capacity with the lowest specific surface area, indicating the importance of the ethanol treatments of biochars produced through the torrefaction conditions—elution of low molecular weight products of torrefaction. In the case of both models, the determination coefficients were high and comparable; however, the lower values of AIC were noted in the case of Freundlich’s isotherms in all types of biochars, indicating that the adsorption had physical characteristics and that the biochar surfaces were not uniform [32,33].

The non-linear regression modeling of Na^+^ adsorption isotherms of HCl-activated biochars indicated a moderate (5-fold) increase in the maximum Na^+^ adsorption capacity in comparison to non-activated biochars (Table 8). The HCl-WSBC had the highest adsorption capacity; and there were also differences between the two other types of HCl-activated biochars. Similarly, as in the case of ethanol activation, the lower values of AIC were noted in the case of Freundlich’s isotherms in all types of biochars. Additionally, the determination coefficients were also higher in Freundlich’s isotherms, and this model should be preferred.

A comparative analysis indicated that in the case of non-activated biochars, the high-temperature pyrolysis biochar should be applied for Na^+^ adsorption. Biochars obtained during low-temperature pyrolysis, mainly torrefaction, are usually not considered as good sorbents of metals due to low sorption capacity, not well-developed specific surface area or, in terms of hard-wood derived biochars, low content of basic cations in exchangeable forms able to replace adsorbents in the solution. However, the results of the study show that to increase Na^+^ adsorption capacity, ethanol activation of a low-temperature biochar can be performed to improve inorganic contaminants sorption capacity. The tests revealed a lower efficiency of HCl treatment in comparison to ethanol. An additional test of ethanol solution composition should be executed, as this aspect of the industrial wastewater production during chemical activation of biochar may create additional problems to be solved with the development of this technology. The presented results concern the initial stage of the technology readiness level development. Additionally, tests aimed at the determination of Na^+^ adsorption optimum conditions (pH, adsorbent dosage as independent values), the investigation of Na^+^ desorption, and the determination of biochar bed breakthrough before the scaling up of this material should be performed.

## 4. Conclusions

The problem of water and soil salinization is a global concern, and there is an urgent need for the development of new, highly efficient, inexpensive and environmentally friendly materials for salt removal. The results of the study showed that waste biomass, such as cereal straw and wood chips, can be converted to biochar, offering a solution for minimizing the agricultural and forestry waste volume and producing valuable Na^+^ adsorbents, reducing the problem of water salinization. Wheat straw biochar, due to its high aromaticity, cation exchange capacity and specific surface area obtained during high-temperature pyrolysis (550 °C), presented the best sorption capacity for Na^+^ removal amongst studied biochars. The tested methods of biochar pre-treatments with EtOH and HCl showed that sorption capacity for Na^+^ can be significantly improved when chemical modifications are applied to biochars produced through torrefaction (<300 °C). The pre-treatment with ethanol of pinewood torrefaction-derived biochar increased the Na^+^ adsorption capacity up to 10-fold compared to non-modified material. The result of the study showed that biochar produced through torrefaction may be utilized for Na^+^ immobilization with lower energy demand and carbon footprint by ethanol treatment, becoming a promising method of material application for environmental and industrial purposes. 

## Figures and Tables

**Table 1:** Changes of cation sorption properties after biochar modification.

Biochar	pH	Ash	CEC	Na^+^	K^+^	Ca^2+^	Mg^2+^
	-	% DM	cmol(+)/kg				
Non-WSBC	9.98 ^1^ ± 0.03 ^2^	36.4 ± 0.6	30.72	27.01 ± 0.2	1.42 ± 0.02	1.43 ± 0.04	0.19 ± 0.02
EtOH-WSBC	9.43 ^1^ ± 0.03 ^2^	19.5	19.67	16.02 ± 0.02	1.11 ± 0.02	1.98 ± 0.02	0.56 ± 0.02
HCl-WSBC	8.89 ± 0.02	33.8	27.71	23.24 ± 0.02	1.15 ± 0.02	2.34 ± 0.02	0.98 ± 0.02

Non-SBC	8.83 ± 0.02	20.3 ± 0.3	3.08	1.13 ± 0.1	1.01 ± 0.01	0.93 ± 0.02	0.11 ± 0.03
EtOH-SBC	8.67 ± 0.02	19.7	3.69	0.98 ± 0.02	1.03 ± 0.02	1.34 ± 0.02	0.34 ± 0.02
HCl-SBC	8.02 ± 0.02	21.0	4.96	1.11 ± 0.02	0.98 ± 0.02	1.98 ± 0.02	0.89 ± 0.02

Non-PBC	6.81 ± 0.02	10.5 ± 0.9	6.64	1.99 ± 0.7	1.45 ± 0.03	2.93 ± 0.01	0.26 ± 0.02
EtOH-PBC	6.63 ± 0.02	7.6	7.31	1.48 ± 0.02	1.94 ± 0.02	3.65 ± 0.02	0.24 ± 0.02
HCl-PBC	6.43 ± 0.02	4.08	8.13	1.78 ± 0.02	1.34 ± 0.02	3.89 ± 0.02	1.12 ± 0.02

^1^ mean values (*n* = 3) ^2^ RSD values (*n* = 3).

**Table 2:** Specific surface area of non-modified and modified biochars.

Biochar	SSA (g/m^2^)
Non-WSBC	265 ^1^ ± 2.1 ^2^_a_
HCl-WSBC	278 ± 3.1 _b_
EtOH-WSBC	387 ± 4.5 _b_
Non-SBC	80.5 ± 2.1 _a_
HCl-SBC	85.4 ± 1.4 _a_
EtOH-SBC	93.2 ± 1.9 _b_
Non-PBC	16.5 ± 1.8 _a_
HCl-PBC	14.5 ± 1.3 _a_
EtOH-PBC	17.8 ± 0.9 _a_

^1^ mean values (*n* = 3) ^2^ RSD values (*n* = 3); _a,b_ Different lowercase letters (a and b) indicate significant differences between non-modified and modified biochars (*p* < 0.05).

**Table 3:** Elemental composition of non-modified and activated biochars and the values of their molar ratios.

Biochar	C	N	H	O	S	C/N	H/C	O/C
	(% w/dw)					(molar)		
Non-WSBC	63.61 ^1^ ± 0.2 ^2^_a_	0.74 ± 0.1 _a_	2.2 ± 0.01 _a_	33.42 ± 0.9 _a_	0.001 ± 0.0 _a_	85.98	0.03	0.52
HCl-WSBC	66.12 ± 0.1 _a_	0.64 ± 0.05 _b_	1.98 ± 0.02 _a_	30.92 ± 1.1 _a_	0.0355 ± 0.01 _b_	104.09	0.02	0.46
EtOH-WSBC	89.42 ± 3.2 _b_	0.63 ± 0.04 _b_	1.81 ± 0.01 _b_	8.11 ± 0.4 _b_	0.0425 ± 0.01 _b_	143.07	0.02	0.09
Non-SBC	79.74 ± 2.1 _a_	0.95 ± 0.1 _a_	2.57 ± 0.03 _a_	16.73 ± 0.3 _a_	0.001 ± 0.03 _a_	83.49	0.03	0.20
HCl-SBC	78.95 ± 1.2 _a_	0.92 ± 0.11 _a_	1.80 ± 0.03 _b_	18.28 ± 1.1 _a_	0.047 ± 0.04 _b_	85.81	0.02	0.23
EtOH-SBC	80.33 ± 1.3 _a_	0.87 ± 0.12 _b_	2.82 ± 0.04 _b_	15.92 ± 0.9 _a_	0.048 ± 0.01 _b_	91.80	0.03	0.19
Non-PBC	89.55 ± 2.7 _a_	0.42 ± 0.03 _a_	2.44 ± 0.01 _a_	7.58 ± 0.3 _a_	0.003 ± 0.01 _a_	213.21	0.03	0.08
HCl-PBC	75.92 ± 2.1 _b_	0.53 ± 0.04 _b_	3.10 ± 0.05 _b_	20.43 ± 0.7 _b_	0.013 ± 0.02 _b_	143.24	0.04	0.26
EtOH-PBC	72.36 ± 3.1 _b_	0.69 ± 0.06 _b_	3.21 ± 0.03 _b_	23.69 ± 0.9 _b_	0.034 ± 0.03 _b_	104.11	0.04	0.32

^1^ mean values (*n* = 3) ^2^ RSD values (*n* = 3); _a, b_ Different lowercase letters (a and b) indicate significant differences between non-modified and modified biochars (*p* < 0.05).

**Table 4:** FT-IR integrated areas of biochars.

Biochar	3188–3720	2800–2989	1480–1660
	–OH	C–H Aliphatic	C–C Aromatic
	% of Area		
Non-WSBC	77.72	0.71	13.90
HCl-WSBC	72.35	1.47	15.04
EtOH-WSBC	71.62	0.96	31.50
Non-SBC	66.91	2.21	13.02
HCl-SBC	82.00	0.56	17.70
EtOH-SBC	85.55	0.87	13.65
Non-PBC	73.83	0.74	15.60
HCl-PBC	56.16	3.93	16.65
EtOH-PBC	63.31	0.97	17.16

**Table 5:** Radical concentration and g-factor calculations for non-modified and modified biochars obtained during EPR analysis.

Biochar	Radical Concentration ×10^−19^ (spin/gram)	g-Parameter
Non-WSBC	0.80	2.0028
HCl-WSBC	0.71	2.0028
EtOH-WSBC	0.42	2.0028
Non-SBC	1.85	2.0028
HCl-SBC	1.88	2.0027
EtOH-SBC	1.20	2.0029
Non-PBC	1.58	2.0029
HCl-PBC	1.63	2.0030
EtOH-PBC	0.46	2.0030

**Table 6:** The comparison of Langmuir’s and Freundlich’s Na adsorption isotherms parameters on non-activated biochars.

Biochar Type	Langmuir’s Model				Freundlich’s Model			
	q_max_, Maximum Adsorption Capacity, mg/g	b, the Ratio of Adsorption Constant Rate to Desorption Constant Rate (*k/k*’)	R^2^—Determination Coefficient	AIC, Akaike Criterion Coefficient	Kf, mg∙g^−1^∙(L∙mg^−1^)^(1/*n*)	(1/*n*), Heterogenicity Coefficient	R^2^—Determination Coefficient	AIC, Akaike Criterion Coefficient
WSBC	308.86	0.00014	0.9909	29.09	0.368	0.678	0.9858	31.13
SBC	245.80	0.00017	0.9724	33.60	0.384	0.657	0.9392	37.50
PBC	214.33	0.00019	0.9932	25.46	0.517	0.614	0.9661	33.51

**Table 7:** The comparison of Langmuir’s and Freundlich’s Na adsorption isotherms parameters on Ethanol-activated biochars.

Biochar Type	Langmuir’s Model				Freundlich’s Model			
	q_max_, Maximum Adsorption Capacity, mg/g	b, the Ratio of Adsorption Constant Rate to Desorption Constant Rate (*k/k*’)	R^2^—Determination Coefficient	AIC, Akaike Criterion Coefficient	Kf, mg∙g^−1^∙(L∙mg^−1^)^(1/*n*)	(1/*n*), Heterogenicity Coefficient	R^2^—Determination Coefficient	AIC, Akaike criterion Coefficient
EtOH-WSBC	2687.32	0.00001	0.9167	42.60	0.002164	1.281	0.9388	41.06
EtOH-SBC	1029.30	0.000028	0.906	42.73	0.009061	1.112	0.925	41.58
EtOH-PBC	3449.54	0.000009	0.9869	33.91	0.021207	1.037	0.9892	32.92

**Table 8:** The comparison of Langmuir’s and Freundlich’s Na^+^ adsorption isotherms parameters on HCl-activated biochars.

Biochar Type	Langmuir’s Model				Freundlich’s Model			
	q_max_, Maximum Adsorption Capacity, mg/g	b, the Ratio of Adsorption Constant Rate to Desorption Constant Rate (*k*/*k’*)	R^2^—Determination Coefficient	AIC, Akaike Criterion Coefficient	Kf, mg∙g^−1^∙(L∙mg^−1^)^(1/*n*)	(1/n), Heterogenicity Coefficient	R^2^—Determination Coefficient	AIC, Akaike Criterion Coefficient
HCl-WSBC	1628.50	0.000059	0.7889	54.09	0.000259	1.707	0.9719	44.04
HCl-SBC	1432.91	0.000068	0.7981	53.83	0.000083	1.858	0.9911	38.21
HCl-PBC	1539.88	0.000063	0.7761	54.48	0.000027	1.999	0.9893	39.31

## Data Availability

Data sharing is not applicable to this article.

## Appendix A

**Table A1:** Samples prepared for adsorption experiment.

Biochar		NaCl (%)				
		0.1	0.2	0.5	1.0	2.0
Non-activated	Non-WSBC	Non-WSBC + 0.1	Non-WSBC + 0.2	Non-WSBC + 0.5	Non-WSBC + 1.0	Non-WSBC + 2.0
	Non-SBC	Non-SBC + 0.1	Non-SBC + 0.2	Non-SBC + 0.5	Non-SBC + 1.0	Non-SBC + 2.0
	Non-PBC	Non-PBC + 0.1	Non-PBC + 0.2	Non-PBC + 0.5	Non-PBC + 1.0	Non-PBC + 2.0
Activated with 0.1 M HCl	HCl-WSBC	HCl-WSBC + 0.1	HCl-WSBC + 0.2	HCl-WSBC + 0.5	HCl-WSBC + 1.0	HCl-WSBC + 2.0
	HCl-SBC	HCl-SBC + 0.1	HCl-SBC + 0.2	HCl-SBC + 0.5	HCl-SBC + 1.0	HCl-SBC + 2.0
	HCl-PBC	HCl-PBC + 0.1	HCl-PBC + 0.2	HCl-PBC + 0.5	HCl-PBC + 1.0	HCl-PBC + 2.0
Activated with 0.1 M C_2_H_5_OH	EtOH-WSBC	EtOH-WSBC + 0.1	EtOH-WSBC + 0.2	EtOH-WSBC + 0.5	EtOH-WSBC + 1.0	EtOH-WSBC + 2.0
	EtOH-SBC	EtOH-SBC + 0.1	EtOH-SBC + 0.2	EtOH-SBC + 0.5	EtOH-SBC + 1.0	EtOH-SBC + 2.0
	EtOH-PBC	EtOH-PBC + 0.1	EtOH-PBC + 0.2	EtOH-PBC + 0.5	EtOH-PBC + 1.0	EtOH-PBC + 2.0

**Table A2:** Concentration of base cations in NaCl solutions used in the experiment.

Solution	Na	Ca	Mg	K
mg/L				
0.1% NaCl	594.3	0.07	0.02	0.09
0.2% NaCl	1188.6	0.12	0.03	0.18
0.5% NaCl	2971.5	0.38	0.04	0.45
1.0% NaCl	5943.2	0.8	0.05	0.9
2.0% NaCl	11,886.5	1.7	0.06	1.8
